# Understanding the support needs of parents of children with obsessive-compulsive disorder: a qualitative descriptive study in the UK

**DOI:** 10.1186/s12888-023-04637-8

**Published:** 2023-05-03

**Authors:** Emma Sowden, Debbie Robinson, Karina Lovell, Penny Bee, Ashley Fulwood, Nicky Lidbetter, Zoe Wilson, Abi Brown, Rebecca Pedley

**Affiliations:** 1 Department of Primary Care and Mental Health, University of Liverpool, 1st Floor Block B Waterhouse Building, Brownlow Street, L69 3GL Liverpool, UK; 2 School of Health Sciences, Division of Nursing, Midwifery and Social Work, Manchester Academic Health Science Centre, University of Manchester, Manchester, UK; 3 School of Health Sciences, Faculty of Biology, Medicine and Health, National Institute for Health and Care Research Applied Research Collaboration Greater Manchester, The University of Manchester, Manchester, UK; 4 OCD UK, 8 Chapel Street, DE56 1AR Belper, Derbyshire, UK; 5 Anxiety UK, Nunes House, 447 Chester Road, Old Trafford, M16 9HA Manchester, UK

**Keywords:** Caregiver burden, Qualitative, Obsessive-compulsive disorder, Carer, Parents, Support, Psychosocial

## Abstract

**Introduction:**

Caring for a child with obsessive-compulsive disorder (OCD) can be extremely difficult, yet evidence-based support strategies for parents/carers are limited. A detailed understanding of parent support needs is an important first step in intervention development and qualitative research with this focus is currently lacking. In this study, the viewpoints of parents and professionals were used to understand support needs and preferences when caring for a child with OCD. This qualitative descriptive study formed part of a wider UK-based project aimed at developing better support for parents of children with OCD.

**Method:**

Individual semi-structured interviews (and an optional one-week journal) with a purposive sample of parents of children and young people (CYP) with OCD, aged 8–18, and focus groups (or individual interviews where preferred) with a purposive sample of professionals supporting CYP with OCD. Data comprised transcripts of audio-recorded interviews and focus groups, and text from journals. Analysis was informed by the Framework approach involving inductive and deductive coding, supported by NVivo 12.0 software. Co-production methods were adopted throughout the research process, including the involvement of a parent co-researcher and charity collaborators.

**Results:**

Interviews were undertaken with 20 parents, of which 16 completed a journal. Twenty-five professionals took part in a focus group or interview. Five key themes relating to parent support challenges and support needs/preferences were identified (1) Coping with the impact of OCD; (2) Getting help for my child; (3) Understanding parents’ role; (4) Making sense of OCD; (5) Joined-up care.

**Conclusion:**

Parents caring for children with OCD have clear caregiver support needs which are currently not being met. Through triangulation of parent and professional accounts, this study has identified parent support challenges (e.g., emotional impact of OCD, visibility of caring role, misunderstanding about OCD) and support needs/ preferences (e.g., headspace/respite, compassion/sensitivity, guidance on accommodation) to lay the vital foundations for the development of effective parent support interventions. There is now an urgent need to develop and test an intervention to support parents in their caregiving role, with the aim of preventing and/or reducing their levels of burden and distress and ultimately, improving their quality of life.

**Supplementary Information:**

The online version contains supplementary material available at 10.1186/s12888-023-04637-8.

## Background

Parental responsibility for a child or young person can be an enjoyable and demanding undertaking [1] that can carry additional challenges when a child develops a mental health difficulty [2, 3]. Without adequate support, this role can have a negative personal, social, and economic impact on individuals providing care [4]. Consequently, a growing body of research has examined the impact of caring for a relative with mental health problems [3, 5–8]. In recent decades, the detrimental impact of caring for a relative with obsessive-compulsive disorder (OCD) has become increasingly recognised [9–17]. As OCD frequently has a paediatric onset, its impact particularly affect carers with parental responsibility [18].

Described as a ‘devastating illness’ that leaves no family member untouched [19], the lives of relatives caring for an individual with OCD are often significantly disrupted [10]. This disruption has been shown to negatively affect carers’ social, working and family lives, as well as their psychological well-being, and quality of life [16, 17, 19, 20]. Carers of people with OCD have also been distinguished from other family carers supporting someone with mental health difficulties owing to how the symptoms can infiltrate and disrupt all aspects of family life [10, 21, 22]. In particular, ‘family accommodation’ is a prevalent feature within families supporting a person with OCD [23], a phenomenon whereby relatives are drawn into supporting the person with OCD with their compulsive rituals, for example, by providing reassurance, modifying daily routines, or helping relatives evade anxiety-provoking situations [14, 21]. Due to children’s natural reliance on their parents for support and reassurance, combined with a parent’s inherent motivation to prevent their child from experiencing distress, parents are viewed as being particularly at risk of accommodating a child’s OCD symptoms [24–27]. Family accommodation [23] is highly prevalent in paediatric OCD and is associated with psychological distress, negative affect, and depression [23, 28–30].

Despite the growing evidence of high levels of burden and distress experienced by parents of children with OCD [24, 31, 32] little attention has been paid to developing evidence-based interventions to support them. This is surprising given the increasing recognition of caregiver burden in the wider health and social care literature [3, 33, 34] and the impetus to support informal carers now highlighted within policy and practice guidelines [35–39]. Only a few published studies have evaluated interventions for parents of children with OCD [40–42]. While these studies report some promising results, these interventions comprise initial examinations including a preliminary evaluation [41], a feasibility study [42] and a quantitative descriptive study involving 26 parents [40]. One intervention consisted of a one-hour educational webinar about OCD [40] and two interventions involved adaptation of existing therapies including mindfulness-based skills training and Quality of Life therapy [41, 42]. Hence there remains a pressing need to establish evidence-based interventions underpinned by detailed intervention development work which is specific to this group of carers.

A detailed understanding of the problem and mechanisms of change which the intervention should target is an important component of intervention development [43, 44]. Much existing research uses quantitative approaches with a focus on measuring the level of burden or impact on quality of life reported by caregivers of relatives with OCD [10, 13, 14, 16] or factors associated with burden or quality of life such as child OCD symptom severity or family accommodation [17, 24, 45]. In addition, many of these studies involve mixed samples of carers, including spouses, adult children, or parents of adult children. Qualitative research with a specific focus on carers with parental responsibility for a child with OCD is limited and has focused on experiences of caring [26, 46], rather than support needs or preferences.

Lazarus and Folkman’s stress-coping model [47, 48] was used as a theoretical model in a quantitative study aimed at understanding family caregiving for adult relatives with OCD in relation to coping strategies [12]. Findings showed that the greater the coping level of family carers, the lesser the social, family, psychological, and spouse relationship burden [12]. While this study provides promise that through increasing parental coping strategies, it may be possible to reduce parent burden and distress, in-depth development work which specifically focuses on parents of children with OCD (not adults with OCD or mixed relatives) is needed to enhance the development of effective support interventions in this area [49].

Given that no previous study to our knowledge has explored parents’, needs and preferences in OCD, qualitative research is particularly appropriate given its strengths in exploring new topics or ideas [50, 51]. Furthermore, multi-perspective interviews and triangulation of accounts have the potential to capture a more nuanced and comprehensive understanding of a phenomenon than can be achieved through a single-perspective approach [52, 53]. This paper will provide a holistic view of parents’ support needs and preferences through the triangulation of viewpoints of parents of children with OCD and professionals working with children with OCD.

We have used the term parent to refer to any adult with parental responsibility for a child or young person (under 18).

## Method

### Design

This qualitative descriptive study [54, 55] was a sub-study within a wider UK-based project entitled Children with OCD: Identifying Acceptable Support Strategies for Parents (CO-ASSIST), which sought to lay the foundations for a support intervention for parents of children with OCD (ISRCTN number:13235264). Specifically, the needs and preferences identified during this qualitative study will be used to inform later stages of the programme where: (i) feasible and acceptable strategies and resources to address parental/carer needs will be agreed with parents and professionals and (ii) the optimal components and content of our intervention will be identified, together with its underpinning theory of change (this work will be reported elsewhere). A qualitative descriptive design, was selected due its appropriacy in informing health related studies where “a straight description of a phenomenon is desired or information is sought to develop and refine questionnaires or intervention”[54] (p.2). Our study was located within a interpretivist frame, which emphasises the importance of gaining an understanding of a phenomenon through people’s interpretations (56, 57). From this perspective, we took into account individual constructions of reality [56].

Parents were invited to participate in semi-structured interviews to explore individual perspectives. They were also given the option of completing a secure electronic journal for seven days leading up to their interview, providing the opportunity to record ‘in the moment’ data, capturing day-to-day challenges and needs. Focus groups (or one-to-one interviews if preferred) were used to explore professionals’ perspectives on parents’ support needs and preferences. As the study was undertaken during the COVID-19 pandemic, to mitigate risk, a fully remote design was employed using video conferencing software (Zoom/Teams) and telephone. The study was conducted in the UK and was given ethical approval by the West of Scotland Research Ethics Committee (Ref:20/WS/0131).

### Co-production research methods

Co-production research methods [58–60) were integral to the study design and included working in partnership with a parent co-researcher (DR) and service-user-led charity representatives (AF, NL, ZW) throughout the research process. DR received training in qualitative research methods and governance, co-facilitated professional focus groups and took a lead role in the analysis and write-up. In addition, the design of the qualitative study was developed through early consultations with parents with experience of caring for a child with OCD (including DR). These early consultations led to the design of an optional parent journal which provided a means of capturing day-to-day challenges and experiences in advance of the interview, adding further depth and richness to the data, and helping parents feel prepared for the interview.

### Sampling and recruitment

As the aim of the wider programme was to inform the development of a support intervention to be evaluated in the United Kingdom, research sites across the UK were eligible. Research sites were identified with the support of research collaborators and the National Institute for Health and Care Research (NIHR) Clinical Research Network (CRN). A range of third sector organisations, including national OCD and anxiety charities, were instrumental in providing recruitment pathways via social media and support channels. Recruitment was conducted between October 2020 and May 2021.

Purposive sampling was used to promote variability in terms of gender, ethnicity, age and stage in the care pathway of the child within the parent sample and discipline & sector for the professional sample. In addition, for parenting couples recruited via NHS sites, individual invitations were sent to each parent separately, to promote the engagement of fathers - a seldom heard group of carers [61, 62]. Potential participants could opt into the study by responding to study adverts posted via social media channels and, at later stages (once Covid-19 restrictions permitted), displayed in NHS clinics. Alternatively, parents could be approached by their child’s direct care team - via personalised letter/email, in-person, or by telephone/video - and professionals by collaborators at participating research sites. Parents received a recruitment pack and were compensated for their time through receipt of a £25 high street gift voucher.

Data saturation is a widely accepted principle in qualitative research used to indicate when data collection should be stopped which should be operationalised in a way that is consistent with the research question and approach used [63, 64]. We viewed saturation as an on-going cumulative judgement amongst the research team regarding the depth of understanding achieved in relation to the developing coding framework rather than a fixed point. [64]. Consensus was reached amongst the research team that sufficient data had been collected to address the research question and that no new information was arising from the data (parent interviews n = 17; professional focus groups n = 4). Three further parent interviews and one focus group were undertaken to confirm that sufficient saturation had been achieved, resulting in a total of 20 parent interviews, 5 professional focus groups and 4 individual professional interviews.

### Data collection

Separate topic guides for parents and professionals were developed by the research team (including experts in OCD, a clinician with expertise in OCD, three OCD/anxiety charity representatives, and a parent co-researcher), based on the aims of the research (Additional file 1). Topic guides included a list of key areas designed to explore participants’ perspectives on parent support needs, however a semi-structured approach allowed the researcher to probe further as new topics and ideas arose. Interviews were conducted by ES, and focus groups were led by three authors (ES, DR & RP). Data was collected by researchers who had no prior relationship with participants. Verbal recorded informed consent was obtained from parents, and electronic informed consent was obtained from professional participants.

### Participants

A total of 20 parents and 25 professionals (see Table 1 for sample characteristics) were recruited from a range of sites in England including: NHS Trusts in England, (based in the North and South-West), four third sector sites (including two national OCD-specific, one national anxiety-specific and one young person’s mental health charity), a multi-agency educational Trust, and social media channels (e.g., online parent support groups, Twitter account, institutional announcements page).

**Table 1:** Sample characteristics (Parental carer and professional participants)

Demographic	Category	N (%)
**Parents (N = 20)**		
Gender	Female	20 *(100)*
Ethnicity	White	20 *(100)*
Age range, years (mean = 47.3)	31–40	2 *(10)*
	41–50	13 *(65)*
	51–60	5 *(25)*
Age of child (mean = 13.8)	8–11	3 *(14)*
	12–15	14 (67)
	16–18	4 *(19)*
Child gender	Male	12 (57)
	Female	9 (43)
Child treatment status	Currently receiving treatment	12 *(57)*
	On waiting list	5 *(24)*
	Not receiving treatment	4 *(19)*
**Professionals (N = 25)**		
**Gender**	Female	20*(80)*
	Male	5 *(20)*
**Profession**	CBT therapists	11 *(44)*
	Clinical Psychologist	3 *(12)*
	Consultant Child & Adolescent Psychiatrist	3 *(12)*
	Mental Health Practitioner	3 *(12)*
	Mental Health Nurse	2 (8)
	Other professional	3 *(12)*

Note: One mother had two children experiencing symptoms of OCD

Twenty parents took part in an individual interview (11 telephone, 9 video), whose families were at various stages of the care pathway. Sixteen completed an online journal. All parents described themselves as female mothers, 19 as White British and one as White American. Twenty-one professionals participated in one of five focus groups, and 4 professionals participated in an individual interview.

### Data analysis

Conversations were digitally recorded, transcribed verbatim, checked for accuracy, and anonymised. The content of the electronic journals was anonymised and placed at the start of the interview transcripts so that each participant’s transcript and journal (where available) could be analysed in parallel. Data analysis was aided by the software NVivo 12.0. Transcripts of interviews/focus groups and the content of journals were analysed using the principles of Framework approach (see Additional file 2) (57). The Framework approach is a flexible thematic approach to analysis which permits both deductive and inductive coding and has been identified as particularly valuable when working with patient and public representatives (providing an experienced qualitative researcher is part of the team) [65]. Due to the lack of previous work in this area, we relied heavily on inductive coding. Owing to our applied health research focus to inform intervention development, deductive coding helped maintain alignment with our research question [66].The theoretical literature on carer burden and stress-coping models were also used as sensitising constructs during coding.

ES and DR led the data analysis, beginning with independent coding of a subset of the data (including eleven parent interviews combined with parent journals and three professional focus groups). Coded data were systematically compared to identify and affirm patterns within the data and inform the initial framework’s development. Convergence of codes across parent and professional data led to the identification of an inclusive framework (following several iterations of framework development), capturing both datasets. ES applied the final framework to the remaining transcripts and charted the data to inform interpretation. The final stage of the analysis involved drafting a manuscript which detailed the identified themes and sub-themes, supported by quotes from the data (selected by ES and DR) to illustrate interpretation.

### Trustworthiness

To ensure trustworthiness of our findings we held regular research team meetings throughout the analytical process to cross-check, discuss and refine the analytical codes and thematic framework. The research team included clinicians, charity representatives, a parent co-researcher, and researchers. Sections of data relating to each theme were shared at team meetings to ensure an agreed interpretation and labelling of codes. Iterations of the developing thematic framework were also shared and refined during team meetings. In addition, we discussed the study process and interim findings with research colleagues outside of the research team.

From an interpretive position, we viewed the researcher as contributing to both the interpretation and construction of knowledge, therefore, to ensure trustworthiness we maintained a process of reflexivity or critical engagement throughout the research process. Reflexive notes were kept regarding the setting and the interview process, and the research team remained critically aware of personal values, preconceptions, and socially derived frames of reference [50]. Throughout the analytical process our parent co-researcher (DR) received training in qualitative research and was supported to engage in a process of reflexivity. Diverse roles and experiences within our multidisciplinary team (including a parent co-researcher, charity partners, OCD researchers and clinicians), were viewed as an important methodological tool to ensure that our interpretation was grounded in parents’ perspectives, while ensuring interpretations stayed close to the data. Triangulation of both data and data collection methods (i.e., interviews, focus groups and journals) across both parents and professionals’ perspectives, enriched the quality and trustworthiness of our findings.

## Results

Our analysis identified five key themes related to support challenges or support needs/preference. Each theme incorporated a related set of 3–4 sub-themes (a total of 18 sub-themes). These sub-themes could be identified as support challenges or support needs/ preferences provided, providing a more nuanced account of the five higher-order themes (Table 2).

The identifier ‘Parent’, followed by an ID number including details of gender, age range, source of the data (journal or interview) has been assigned to all parents’ quotes. The identifier ‘Professional’ followed by an ID number, including gender and sources of data- Focus Group Discussion (FGD) or interview has been assigned to all professional quotes.

**Table 2:** Thematic framework illustrating parent support challenges and support needs/preferences

Theme	Sub-theme	
	Support challenges	Support needs/preferences
**Coping with the impact of OCD**	Overwhelming nature of OCD	Headspace (respite)
		Compassion & sensitivity
		Sharing experiences
**Getting help for my child**	A battle to access OCD treatment	Being heard by professionals
		Firming up the OCD pathway
**Understanding parents’ role**	Uncertainty & confusion about accommodation	Guidance on accommodation
	Visibility of caring role	Treatment expectations
**Making sense of OCD**	Confusing nature of OCD	Parent-focused information
	OCD and other conditions	
**Joined-up care**	Misunderstandings about OCD	A united approach
	Lack of shared understanding in the family	
	Lack of coordination & unity across & within services	

### Coping with the impact of OCD

#### Support challenge: the overwhelming nature of OCD

Parents described experiencing a range of emotions as they dealt with the overwhelming demands of supporting a child with OCD. Witnessing the negative impact of OCD on family life resulted in parents feeling guilt, blame, sadness, and exhaustion:

*“Feeling of exhaustion, anger, sad, guilty, and tired. Just sick of OCD ruling our lives.”* (Parent 6: Journal entry, female age 50–54).

Caring responsibilities such as liaising with school and health services were compounded by extra undertakings controlled by their child’s symptoms of OCD, including extra shopping, cleaning, washing, purchasing special food or household items, disturbed sleep resulting in an immense disruption to family life:

*“…It has taken over our entire life and everyone’s life in the house and affected everything.”* (Parent 16: interview, female, age 45–49).

#### Support need/preference: headspace (respite)

Both parents and professionals recognised the need for parents to receive ‘headspace’ or respite from their caring role, a responsibility conveyed as relentless and all-consuming:

*“It’s that relentless 24/7 pressure, I think, that is what we’re seeing, isn’t it? Why they’re struggling so much, it’s just that lack of having any break.”* (Professional 10, FGD 3, Female).

Parents who had coped for extended periods without a break seemed unclear about what would help. Others were unclear about how to create this space for themselves due to the unrelenting demands of caring for a child with OCD.

#### Support/need preference: compassion & sensitivity

Both professionals and parents recognised compassion, understanding and sensitivity regarding what families had to deal with as supportive and encouraging for parents. Conversely, a lack of kindness or empathy could leave parents feeling alone, unsupported, and unvalued. The following parent spoke of the positive difference compassion and sensitivity made when it was eventually encountered:

*“The first person who was helpful and showed compassion. She admitted she didn’t know anything about OCD and spent a lot of time on the phone letting me explain. Felt supported and that she was genuinely interested.”* (Parent 1: journal entry, female, age 45–49).

Parents also valued sensitivity and understanding from professionals when discussing their role in managing their child’s symptoms to help counter feelings of blame or guilt. Professionals also discussed the need to help families attribute the symptoms of OCD to an external source (e.g., conceptualising OCD as a ‘bully’) to help alleviate any feelings of blame or guilt a parent may be experiencing.

#### Support/need preference: sharing experiences

To counter what was often conveyed as an isolating and frightening journey for parents, the value of parents having the opportunity to talk to people who understand such as other people who had gone through similar experiences or ‘non-judgemental’ professionals who had time to listen, was emphasised within both parent and professional accounts.

*“I would like to speak to other people who experience it, the same, or similar sort of things as* [name of son]. *Because I think, as a parent, it is quite frightening, yeah, it is very frightening…”* (Parent 5: interview, female, age 45–49).

Some parents and professionals indicated a preference for in-person parent support groups with a potential for these being specifically aimed at parents of children with OCD. Nonetheless, potential difficulties were identified such as limited resources, and a need for flexibility (owing to carer and parental responsibilities).

### Getting help for my child

#### Support challenge: a battle to access OCD treatment

Parents and professional accounts indicated that a parent’s overwhelming priority was to get the right help for their child, which parents frequently depicted as a battle involving pushing, fighting and which required extensive energy, resources, and determination:

*“…if you are proactive and you push and fight and fight and fight, you will finally start to get some help, and that is the problem”* (Parent 9: interview, female, age 40–44).

Both parents and professional indicated that parents often neglected their own needs in their commitment and dedication to getting help for their child. Establishing a diagnosis of OCD was often conveyed by both parents and professionals as a lengthy process which could be complicated by misdiagnosis, lack of specialist assessment, the presence of co-existing conditions or long waiting lists. Parents described feeling desperate and uncertain, often having to access alternative services during this period, including emergency mental health services, Accident and Emergency, private therapists and GPs. Long waiting lists and a lack of parity between how physical and mental health conditions were resourced resulted in frustration for parents and professionals:

*“…if they had diabetes or something like that. You wouldn’t say, oh, we’ll wait until you have a diabetic crisis before we start really getting involved and stuck in here; we’ll do it right at the very front end.”* (Professional 17, FGD 5, male).

When families finally managed to access the correct treatment, the relief brought by improvement in their child’s condition could be dampened by the regret of lost years living with the debilitating impact of OCD whilst waiting for treatment.

#### Support need/preference: being heard by professionals

Having their concerns heard and taken seriously was a key priority for parents, yet many felt they were not listened to as they tried to seek professional help for their child:

*“I just felt like I was banging my head on a brick wall, and nobody was really listening, and as his mum, I knew what was happening to him, but I was powerless to help him”* (Parent 17: interview, female, age 40–44).

Descriptions of waiting until a crisis point to be heard or noticed were evident in parent and professional accounts.

### Support need/preference: firming up the OCD pathway

Professionals and parents highlighted a need to strengthen or ‘firm up’ the OCD pathway to provide timely access to evidence-based treatment and to avoid families going down the wrong route:

*“I think there needs to be actual evidence-based treatment and interventions. Not someone having a nice chat, person-centred counselling does not touch OCD. We need to stop sending children with OCD who don’t meet CAMHS thresholds to another service for person-centred help, which is not the right treatment.”* (Professional 6: interview, female).

While the ideal situation was viewed as timely evidence-based treatment for OCD, there was widespread recognition of the need to have access to appropriate resources whilst on a waiting list, with the caveat that these resources must not become an alternative but rather complementary to the service.

### Understanding parents’ role

#### Support challenge: visibility of caring role

Findings illustrated how parents struggled to balance multiple roles, including parent, carer, and co-therapist, whilst maintaining employment and wider family relationships. Yet there was little recognition and support for what seemed to be their most challenging role, caring for their child with OCD. This pervading theme was captured effectively by the quote from a professional below:

*“…I don’t think that societally we’ve got a system which recognises parents, that if it were an adult with that level of need that there would be benefits, there would be things; but I think that when it’s a child, the expectation is you* [parents] *just crack on and look after them and get on with it.”* (Professional 9: FGD 3, male).

The space to consider parent needs through taking part in this study provided validation and was appreciated by individuals within this caring role. The gap in parent focused support was widely acknowledged. However, professional role boundaries meant there was limited space, resources or expertise within child treatment protocols to consider parents’ support needs in their own right. Furthermore, signposting to outside resources was variable.

#### Support challenge: uncertainty & confusion around accommodation

Parents were frequently unaware they had been accommodating and regretted not being provided with advice or guidance earlier to prevent the symptoms of OCD from becoming entangled within family life. An individual’s discovery that what they perceived as doing the best for their child may have been making their child’s symptoms worse was distressing, and some parents described feeling as though they had been reprimanded or blamed by the professional:

*“At times, I’ve felt like I’ve been, had my wrists slapped for accommodating things, which hasn’t felt very nice. I guess, you know, any personal criticism about the way you think that you’re supporting your child to the best and then to find out that actually you’ve possibly been fuelling the problem was quite a distressing moment.”* (Parent 4: interview, female, age 45–49).

Parent accounts suggested that defining the boundaries between caring and accommodating was challenging. This was particularly exaggerated with the co-occurrence of other conditions, where differentiation between conditions could be problematic and approaches to treatment less clear.

#### Support/need preference: guidance on accommodation

Parents and professionals discussed the difficult, emotionally taxing task faced by parents when trying to withdraw accommodation during treatment. Despite recognising parents’ struggles, professionals felt limited in their capacity to support parents and emphasised a need for more resources to support and guide parents during this challenging process:

*“As a clinician, you feel bad, don’t you, trying to convince them* [parents] *not to do those things. It would really enhance that experience if it felt that it was supported by wider voices.”* (Professional 10: FGD 3, female).

#### Support need/preference: treatment expectations

Parents described how at times, they had felt despairing about their child’s condition and emphasised the need for parents to be informed that OCD is a treatable condition as early in the journey as possible:

*“…if someone could say it’s not the end of the world and there is help out there because, at the time when it all got very serious, it just feels like the end of the world, your child is never going to overcome it, never get better.”* (Parent 17: interview, female, age 40–44)

Professionals acknowledged that parents’ key concerns were around their child’s recovery and uncertain future and that hope was an important message to counter this distress.

An honest and open dialogue about treatment expectations was viewed as essential to foster parents’ understanding and conviction in the treatment process, which was portrayed as a hard journey that required extensive work and commitment:

*“I guess the message is that a lot of people with OCD do get better, but it takes a while, and it will require quite a lot of effort. So, it’s not taking away the hope, but then I guess it’s being realistic...” (*Professional 9: FGD,3 male)

### Making sense of OCD

#### Support challenge: confusing nature of OCD

OCD was viewed by parents as a tricky condition to understand due to its shifting presentations and how it could unwittingly or explicitly cajole, coerce, or bully family members’ involvement. Professionals also recognised the difficulties faced by parents when trying to understand OCD:

*“I think you have to understand about it because it is so sophisticated in its kind of bullying, and it’s so complex. And I don’t think your average person really understands that something could take your values and then turn them on its head and that your brain would do that to you.”* (Parent 5: interview, female, age 45–49)

Presentations of OCD, which did not often map onto stereotypes of the condition (including thoughts around harm or sexually intrusive thoughts or where their child’s ritual involved seeking reassurance), left some parents feeling distressed and confused about what was wrong with their child:

“.*when young people present with those thoughts around harm and sexually intrusive thoughts, you know, if families are maybe not realising that that is OCD, then that can cause a lot of difficulties just because of that lack of understanding…”* (Professional 5: FGD 1, female).

#### Support challenge: OCD & other conditions

Parents and professionals described how when OCD co-existed with other health conditions, understanding and treatment could be more challenging without adapted resources:

*“They actually went and got a book, they had the book in front of them, and they’re saying, well, this is how we do CBT. I was like, but he’s not at that level. He’s got autism; he doesn’t understand what you’re saying to him. So, they didn’t adapt anything. The language or anything, adaptations for him.”* (Parent 6: interview, female, age 50–54).

#### Support need/preference: parent-focused information

Parents and professionals viewed educational support and resources to help parents make sense of OCD and its treatment, as an important resource required to support parents. Yet, accounts showed that the provision of informational resources and educational support was limited and where such provision was available, acceptability was limited. Accessible information or resources specifically designed for parents (rather than the individual experiencing OCD), were prioritised by parents:

*“The audience for all of this stuff is the person with OCD. I think what they need is some information geared towards the audience of the people who are supporting them that don’t have OCD because we’re coming from it from a completely different angle.”* (Parent 19: interview, female, age 40–44).

### Joined up care

#### Support challenge: misunderstandings about OCD

It was felt by most participants that members of the public and even many professionals, failed to recognise OCD as a serious and debilitating mental health condition:

*“I know society will joke about OCD, but unless you’re living with it, I don’t think you can have any idea as to how controlling it is really of your life.”* (Parent 12: interview, female, age 40–44).

Concern was expressed about the negative impact of societal misrepresentation of OCD as this could lead to parents feeling confused by the debilitating nature of the condition and significant impact on the family. Both parents and professionals described coming into contact with practitioners who lacked understanding of and expertise in OCD, which could hinder effective care.

#### Support challenge: lack of shared understanding in the family

Parents and professional accounts conveyed how a child often engaged differently with each family member, and discrepancies could occur between parents’ understanding and approach to caring, which could exacerbate the disruption caused by a child’s OCD symptoms:

*“My husband and I, you know, were at loggerheads about how we were dealing with* [our daughter’s] *challenging behaviour”* (Parent 4: interview, female, age 45–49).

#### Support challenge: lack of coordination & unity across & within services

Parents and professionals recognised that services that lacked awareness or that did not coordinate effectively (particularly across school and mental health services), presented challenges for families. Owing to the centrality of school to a child’s life, parents and professionals recognised the significance of a supportive school; however, accounts indicated that communication and support from schools was variable:

*“I have tried to engage with the school, but they just don’t get it. The school don’t have the skill, knowledge or resources to support this kind of condition. School is a big challenge. Teachers are not skilled in these areas and don’t have the resources and capacity to deal with it. It is a constant fight to get the right person”.* (Parent 1: Journal entry, female, age 45–49)

#### Support need/preference: a united approach

A united approach across services and within the family was conveyed as having the potential to ameliorate the challenges and lessen the demands the symptoms of OCD can create:

*“… if we’ve got a united front … then we stand a chance of all of us standing up against OCD.”* (Professional 6: interview, female).

Accounts indicated a need for a well-informed multi-agency network extending to professions who may encounter parents earlier on in their journey or may act as parents’ first point of contact (e.g., schools, GPs and professionals involved in CAMHS assessment) to ensure families were adequately supported throughout their journey as a carer.

## Discussion

This study used a descriptive qualitative approach involving parents and professionals to gain a holistic understanding of parent, support needs and preferences, when caring for a CYP with OCD. To our knowledge, this is the first qualitative study to have specifically investigated support needs in parents of children with OCD, from either a parent or professional perspective. High levels of parental strain, distress and burden were evident throughout parent accounts, as they tried to support their child with OCD, generally with little or no support.

The five themes identified and presented in this paper, have implications for future intervention development and policy direction in this area. Within each of these themes, a comprehensive understanding of the key support challenges (9 sub-themes) and parents’ needs/preferences for support (9 sub-themes) were identified. Resonating with existing literature, findings showed high levels of burden and distress associated with caring for a child with OCD [13, 24, 67]. Yet, in contrast to other qualitative research in this area [26, 46] we were able to provide insights into how system-level or structural factors such as difficulty accessing OCD treatment for their child, misunderstandings about OCD, lack of visibility of parents’ as carers and variability in joined-up care, could act as potential barriers to optimal caregiver support.

Without adequate carer support or guidance, parents reported having become involved in their child’s core OCD symptoms to try and mitigate their child’s distress. Consequently, this lack of appropriate support at the right time often resulted in parents feeling guilty and regretful that they could not access the correct treatment for their child or had not been given earlier advice on how best to support their child. Professionals’ accounts resonated with those of parents. They empathised with the parents’ position and expressed discomfort and frustration at the lack of timely access to evidence-based treatment for children and in not being able to provide more support addressing parents’ needs as carers within the constraints of child treatment protocol, which together they knew to have detrimental consequences on the family.

Parents’ recognition of themselves as a carer with their own caring needs was complicated by role-expectations associated with parental responsibility. This was most evident within the key theme ‘getting help for my child’. Above everything else, getting the right treatment for their child was viewed as a parents’ priority. Parents within this sample unquestionably sacrificed their own needs in their commitment to support their child. Barriers to timely access to appropriate therapeutic support for their child were frequent and distressing for parents, who could not consider their own needs until this was addressed. This process was frequently viewed as a battle, which required constant pushing and fighting which has been previously reported in the literature in explorations of parents’ experiences of accessing mental health services for child anxiety problems [68]. This experience resulted in feelings of exhaustion and burden and has implications for where a parent resource would be optimally positioned.

Within this fraught context, parents were not always able to recognise the necessity of prioritising their own support needs as a carer to sustain their well-being. These findings resonate with existing literature that indicates this group of caregivers perceive a greater responsibility in ensuring treatment progress for their child with OCD compared to other family carers such as spouses [10, 69].

Parents and professionals valued the opportunity to speak about parents’ caring role, which was often overlooked amidst parental responsibility and a parents’ role in their child’s treatment protocol. The lack of visibility of this caring role amidst other responsibilities was significant given the widespread policy, guidelines, and commitments to support carers [37, 70]. These findings indicate a need for policy and service development to advance evidence-based parent support interventions and raise the profile of this underserved group of carers. Giving parents permission to recognise themselves as carers and feel validated to consider their needs is an essential consideration for future policies and guidelines linked to paediatric OCD. Also noticeable within parents and professional accounts was the lack of use of the term ‘burden’ or the fit of this term within a parental context. The literature has previously indicated a growing discomfort with the term ‘burden’, which ignores the role’s contextual, affective and relational elements that may be most central to caregivers [71].

Unsurprisingly, within this challenging context, where the child’s needs were often at the forefront of priorities, parents and professionals could not always identify specific parent support strategies, however across accounts, broader areas of parent support needs and preferences were identified. These included potential parent-level support/needs preferences that could be considered for future support intervention, including a need for headspace (respite), compassion and sensitivity, guidance on accommodation, treatment expectations, and parent-focused information. Some of these preferences and support needs resonated with findings in the wider literature, such as the sub-theme within our data, ‘sharing experiences’ which could be translated into the value of peer support. Peer support is based on the premise that individuals who have gone through the same difficulties can make an interpersonal connection on that basis and can come together and understand each other’s distress and share skills and knowledge [72, 73]. The value of parent to parent peer support [74, 75] and peer support in mental health services [76, 77] has been recognised in the literature. Ensuring peer support is delivered flexibly and situated outside of the resource constraints of mental health services, so parents can share learning as early as possible, are important considerations to take forward in intervention development work.

Compassion was integral to parents feeling supported and expressions of compassion were highly valued by parents, conveyed as uplifting but potentially constrained by limited resources. Parents particularly valued sensitivity and compassion with regards to the overwhelming emotional nature of caring for a child with OCD, and feelings of guilt or regret concerning their child’s onset of OCD and confusion around accommodation. While compassion is viewed as a hallmark of high quality services, our findings support the view that compassion should not be viewed as a straightforward solution consigned to individual healthcare professionals but something that needs to be understood in relation to the context, energy, understanding and resources in which compassionate care is being delivered [78].

The existence of constraints in child mental services, visibility of the carer role and the benefits of early guidance on accommodation, highlighted the potential value of integrated access to parent/carer-focused support interventions early in a parents’ journeys (such as schools and primary care services). Nonetheless, accounts indicated the significance of ensuring that parent support should be complementary to and not an alternative to adequate evidence-based child services.

### Strengths and limitations

Most professionals in this study had specialist training or a particular interest in OCD treatment, so their views may not represent the broader population of professionals who may encounter children with OCD. In addition, our sample comprised parents identifying as female, so we have not represented other gender identities within our findings. Despite purposively sampling for ethnicity, all parents identified as white, so the applicability of the findings to parents of other ethnicities requires further research. Although, we recruited through national charities which meant that the study was open to people in other nations of the UK, all participants resided in England so transferability of our findings to other UK nations may be limited (for example, due to cultural differences between nations and differences in health services).

The adoption of a fully remote design during the pandemic prompted a departure from our plans to use a face-to-face interview design which was supplemented by some remote data collection. While the use of solely remote data collection methods (telephone and video) rather than face to face could have affected the quality of data (e.g., through a decrease in non-verbal cues), changing our study to a fully remote design meant that we could generate a wide geographical spread of participants, ensuring that accounts were not too heavily influenced by local factors, (e.g. service provision in a certain area) and maximising accessibility for participants.

Our initial focus was to sample children (ages 8–11) and young people (ages 12–18) by asking sites to target age groups. However, feedback from clinicians and parents indicated that younger children are typically less likely to be diagnosed with OCD, so our sample of younger children was small owing to a limited recruitment pool. Nonetheless, we captured some age variability, and parents often reflected on earlier experiences when their child was younger, before OCD was formally identified. A key strength of the study has been the adoption of co-production research methods involving close collaborative with our parent co-researcher and charity partners throughout the research process, enhancing the sensitivity and quality of our approach and the credibility and trustworthiness of our findings.

## Conclusion

The current gap in the provision to support parents as carers of children with OCD in their own right, combined with strong support for intervention development was evident. Parent-level needs and preferences, including headspace, respite, compassion and sensitivity, sharing experiences, guidance on accommodation, treatment expectations, and parent-focused information identified within this study, have the potential to inform components of a parent focused support intervention. Barriers to getting the right help for their child, lack of visibility as a carer, misunderstandings about OCD, and insufficient joined-up care were evidence of the need to consider wider organisational, policy and public health issues when developing an intervention for this group of carers in the UK. In addition to indicating a need for parent-level interventions, this research has highlighted the need to improve access to specialist OCD treatments for children and the need to improve public understanding of OCD. The findings also highlighted the value of a co-produced, person-centred intervention in which the needs and preferences of those for whom the resource is intended are prioritised. Our team aims to address this longer-term goal through a wider programme of research to develop and test an intervention to support parents in their caregiving role, with the aim of preventing and/or reducing their levels of burden and distress and ultimately, improving their quality of life.

## Electronic supplementary material

Below is the link to the electronic supplementary material.

Supplementary Material 1

Supplementary Material 2

## Data Availability

The dataset generated and analysed during this study are not publicly available due to a potential breach of anonymity but are available from the corresponding author on reasonable request.

## Abbreviations

CAMHS: Child Adolescent Mental Health Services
CBT: Cognitive behaviour therapy
CRN: Clinical Research Network
CYP: Children and Young people
GP: General Practitioner
OCD: Obsessive-compulsive disorder
NHS: National Health Service
NIHR: National Institute for Health and Care Research.

## References

[1] Blustein J (2012). Doing the best for one’s child: satisficing versus optimizing parentalism. Theor Med Bioeth.

[2] Liu M, Lambert CE, Lambert VA (2007). Caregiver burden and coping patterns of chinese parents of a child with a mental illness. Int J Ment Health Nurs.

[3] Meltzer H, Ford T, Goodman R, Vostanis P (2011). The burden of caring for children with emotional or conduct disorders. Int J Family Med.

[4] Mendenhall AN, Mount K (2018). Parents of children with Mental Illness: exploring the Caregiver experience and caregiver-focused interventions. Families in Society: The Journal of Contemporary Social Services.

[5] Awad AG, Voruganti LN (2008). The burden of schizophrenia on caregivers: a review. PharmacoEconomics.

[6] Hoenig J, Hamilton MW (1966). The schizophrenic patient in the community and his Effect on the Household. Int J Soc Psychiatry.

[7] Maurin JT, Boyd CB (1990). Burden of mental illness on the family: a critical review. Arch Psychiatr Nurs.

[8] Mittendorfer-Rutz E, Rahman S, Tanskanen A, Majak M, Mehtälä J, Hoti F (2019). Burden for parents of patients with Schizophrenia-A Nationwide Comparative Study of parents of offspring with rheumatoid arthritis, multiple sclerosis, Epilepsy, and healthy controls. Schizophr Bull.

[9] Kalra H, Kamath P, Trivedi JK, Janca A (2008). Caregiver burden in anxiety disorders. Curr Opin Psychiatry.

[10] Cooper M (1996). Obsessive-compulsive disorder: effects on family members. Am J Orthopsychiatry.

[11] Black D, Gaffney G, Schlosser S, Gabel J (1998). The impact of obsessive-compulsive disorder on the family: preliminary findings. J Nerv Mental Disease.

[12] El-Slamon MAEA, Al-Moteri M, Plummer V, Alkarani AS, Ahmed MG. Coping Strategies and Burden Dimensions of Family Caregivers for People Diagnosed with Obsessive-Compulsive Disorder. Healthcare. 2022 Feb 28;10(3):451. 10.3390/healthcare10030451 PMC8954481 35326929

[13] Grover S, Dutt A (2011). Perceived burden and quality of life of caregivers in obsessive-compulsive disorder. J Neuropsychiatry Clin Neurosci.

[14] Laidlaw TM, Falloon IR, Barnfather D, Coverdale JH (1999). The stress of caring for people with obsessive compulsive disorders. Community Ment Health J.

[15] Ramos-Cerqueira AT, Torres AR, Torresan RC, Negreiros AP, Vitorino CN (2008). Emotional burden in caregivers of patients with obsessive-compulsive disorder. Depress Anxiety.

[16] Stengler-Wenzke K, Kroll M, Matschinger H, Angermeyer MC (2006). Quality of life of relatives of patients with obsessive-compulsive disorder. Compr Psychiatry.

[17] Sücüllüoğlu Dikici D, Eser E, Çökmüş FP, Demet MM (2019). Quality of life and associated risk factors in caregivers of patients with obsessive compulsive disorder. Psychiatry and Clinical Psychopharmacology.

[18] Geller DA, Homayoun S, Johnson G. Developmental Considerations in Obsessive Compulsive Disorder: Comparing Pediatric and Adult-Onset Cases.Frontiers in Psychiatry. 2021;12. 10.3389/fpsyt.2021.678538 PMC8269156 34248714

[19] Soni S, C P, Ahamed A. Understanding and Management of Caregivers’ Stress and Burden of Person with Obsessive Compulsive Disorder. 2017.

[20] Albert U, Salvi V, Saracco P, Bogetto F, Maina G (2007). Health-related quality of life among first-degree relatives of patients with obsessive-compulsive disorder in Italy. Psychiatric Serv (Washington DC).

[21] Calvocoressi L, Mazure CM, Kasl SV, Skolnick J, Fisk DA, Vegso SJ (1999). Family accommodation of obsessive-compulsive symptoms: instrument development and assessment of family behavior. J Nerv Ment Dis.

[22] Oza H, Parikh MN, Vankar GK (2017). Comparison of caregiver burden in schizophrenia and obsessive–compulsive disorder. Arch Psychiatry Psychother.

[23] Lebowitz ER, Panza KE, Bloch MH (2016). Family accommodation in obsessive-compulsive and anxiety disorders: a five-year update. Expert Rev Neurother.

[24] Wu MS, Hamblin R, Nadeau J, Simmons J, Smith A, Wilson M (2018). Quality of life and burden in caregivers of youth with obsessive-compulsive disorder presenting for intensive treatment. Compr Psychiatry.

[25] Lebowitz ER, Omer H, Leckman JF (2011). Coercive and disruptive behaviors in pediatric obsessive-compulsive disorder. Depress Anxiety.

[26] Futh A, Simonds LM, Micali N (2012). Obsessive-compulsive disorder in children and adolescents: parental understanding, accommodation, coping and distress. J Anxiety Disord.

[27] Freeman JB, Garcia AM, Fucci C, Karitani M, Miller L, Leonard HL (2003). Family-based treatment of early-onset obsessive-compulsive disorder. J Child Adolesc Psychopharmacol.

[28] Albert U, Baffa A, Maina G (2017). Family accommodation in adult obsessive-compulsive disorder: clinical perspectives. Psychol Res Behav Manage.

[29] Storch EA, Geffken GR, Merlo LJ, Jacob ML, Murphy TK, Goodman WK (2007). Family Accommodation in Pediatric Obsessive–Compulsive disorder. J Clin Child Adolesc Psychol.

[30] Pontillo M, Demaria F, Tata MC, Averna R, Gargiullo P, Pucciarini ML (2020). Clinical significance of family accommodation and parental psychological distress in a sample of children and adolescents with obsessive-compulsive disorder aged 8–17 years old. Ital J Pediatr.

[31] Stewart SE, Hu YP, Leung A, Chan E, Hezel DM, Lin SY (2017). A Multisite Study of Family Functioning Impairment in Pediatric Obsessive-Compulsive disorder. J Am Acad Child Adolesc Psychiatry.

[32] Amir N, Freshman M, Foa EB (2000). Family distress and involvement in relatives of obsessive-compulsive disorder patients. J Anxiety Disord.

[33] Bastawrous M (2013). Caregiver burden–a critical discussion. Int J Nurs Stud.

[34] Baronet AM (1999). Factors associated with caregiver burden in mental illness: a critical review of the research literature. Clin Psychol Rev.

[35] Alderwick H, Gardner T, Mays N (2021). England’s new health and care bill. BMJ.

[36] Great B (2014). Care Act 2014. Chapter 23.

[37] Medical directorate and Nursing directorate. NHS England’s Commitment for Carers. In: England N, editor. 2014.

[38] Freedom PsN, Health CoM. Achieving the Promise: Transforming Mental Health Care in America Rockville (MD): Department of Helth and Human Resources. 2003 [Available from: https://govinfo.library.unt.edu/mentalhealthcommission/reports/reports.htm.

[39] National Institute for Health and Clinical Excellence (NICE) (2005). Obsessive–compulsive disorder: Core Interventions in the treatment of obsessive–compulsive disorder and body dysmorphic disorder (clinical Guideline 31).

[40] Jassi AD, Kolvenbach S, Heyman I, Macleod T, Rose J, Diamond H (2016). Increasing knowledge about obsessive compulsive disorder and support for parents and schools: evaluation of initiatives. Health Educ J.

[41] Abedi MR, Vostanis P (2010). Evaluation of quality of life therapy for parents of children with obsessive-compulsive disorders in Iran. Eur Child Adolesc Psychiatry.

[42] Belschner L, Lin SY, Yamin DF, Best JR, Edalati K, McDermid J (2020). Mindfulness-based skills training group for parents of obsessive-compulsive disorder-affected children: a caregiver-focused intervention. Complement Ther Clin Pract.

[43] Skivington K, Matthews L, Simpson SA, Craig P, Baird J, Blazeby JM (2021). A new framework for developing and evaluating complex interventions: update of Medical Research Council guidance. BMJ.

[44] Yardley L, Morrison L, Bradbury K, Muller I (2015). The person-based Approach to intervention development: application to Digital Health-Related Behavior Change Interventions. J Med Internet Res.

[45] Suculluoglu-Dikici D, Cokmus FP, Akin F, Eser E, Demet MM (2020). Disease burden and associated factors in caregivers of patients with obsessive-compulsive disorder. Dusunen Adam.

[46] Lebowitz ER, Vitulano LA, Omer H (2011). Coercive and disruptive behaviors in pediatric obsessive compulsive disorder: a qualitative analysis. Psychiatry: Interpers Biol Processes.

[47] Lazarus RS, Folkman S (1984). Stress, Appraisal, and coping.

[48] Folkman S, Lazarus RS, Dunkel-Schetter C, DeLongis A, Gruen RJ (1986). Dynamics of a stressful encounter: cognitive appraisal, coping, and encounter outcomes. J Pers Soc Psychol.

[49] Hoagwood KE, Cavaleri MA, Serene Olin S, Burns BJ, Slaton E, Gruttadaro D (2010). Family support in children’s mental health: a review and synthesis. Clin Child Fam Psychol Rev.

[50] Mason J. Qualitative researching: Sage; 2017.

[51] Merriam SB, Tisdell EJ. Qualitative research: a guide to design and implementation. John Wiley & Sons; 2015.

[52] Kendall M, Murray SA, Carduff E, Worth A, Harris F, Lloyd A et al. Use of multiperspective qualitative interviews to understand patients’ and carers’ beliefs, experiences, and needs. BMJ. 2009;339. 10.1136/bmj.b4122 19828645

[53] Vogl S, Schmidt E-M, Zartler U (2019). Triangulating perspectives: ontology and epistemology in the analysis of qualitative multiple perspective interviews. Int J Soc Res Methodol.

[54] Kim H, Sefcik JS, Bradway C (2017). Characteristics of qualitative descriptive studies: a systematic review. Res Nurs Health.

[55] Sandelowski M (2010). What’s in a name? Qualitative description revisited. Res Nurs Health.

[56] Given LM. (Ed.) The SAGE encyclopedia of qualitative research methods. Sage Publications; 2008.

[57] Ritchie J, Lewis J, Nicholls CM, Ormston R. Qualitative research practice: A guide for social science students and researchers: Sage; 2013.

[58] Farr M, Davies R, Davies P, Bagnall D, Brangan E, Andrews H. A map of resources for co-producing research in health and social care. National Institute for Health Research ARC West and People in Health West of England. 2020.

[59] Coldham T, Group I. Guidance on co-producing a research project. National Institute for Health Research UK; 2018.

[60] Hickey G, Brearley S, Coldham T, Denegri S, Green G, Staniszewska S (2018). Guidance on co-producing a research project.

[61] Dolan A (2014). ‘I’ve learnt what a dad should do’: the interaction of masculine and fathering identities among men who attended a ‘dads only’parenting programme. Sociology.

[62] Scourfield J, Allely C, Coffey A, Yates P (2016). Working with fathers of at-risk children: insights from a qualitative process evaluation of an intensive group-based intervention. Child Youth Serv Rev.

[63] Fusch P, Ness LR (2015). Are we there yet? Data saturation in qualitative research. Qualitative Rep.

[64] Saunders B, Sim J, Kingstone T, Baker S, Waterfield J, Bartlam B (2018). Saturation in qualitative research: exploring its conceptualization and operationalization. Qual Quant.

[65] Gale NK, Heath G, Cameron E, Rashid S, Redwood S (2013). Using the framework method for the analysis of qualitative data in multi-disciplinary health research. BMC Med Res Methodol.

[66] Bingham AJ, Witkowsky P, Vanover C, Saldaña J (2022). Deductive and inductive approaches to qualitative data analysis. Analyzing and interpreting qualitative data: after the intervie.

[67] Storch E, Lehmkuhl H, Pence S, Geffken G, Ricketts E, Storch J (2008). Parental experiences of having a child with obsessive-compulsive disorder: Associations with clinical characteristics and Caregiver Adjustment. J Child Fam stud.

[68] Crouch L, Reardon T, Farrington A, Glover F, Creswell C (2019). “Just keep pushing”: parents’ experiences of accessing child and adolescent mental health services for child anxiety problems. Child Care Health Dev.

[69] Stengler-Wenzke K, Trosbach J, Dietrich S, Angermeyer MC (2004). Coping strategies used by the relatives of people with obsessive-compulsive disorder. J Adv Nurs.

[70] Woods R, McCormick S. Carer Wellbeing and Supports: A review of the literature and directions for research, Centre for Carers Research, Institute of Public Policy and Governance, University of Technology Sydney. 2018.

[71] Sales E (2003). Family burden and quality of life. Qual Life Res.

[72] Stratford AC, Halpin M, Phillips K, Skerritt F, Beales A, Cheng V (2019). The growth of peer support: an international charter. J Mental Health.

[73] Gillard S (2019). Peer support in mental health services: where is the research taking us, and do we want to go there?. J Mental Health.

[74] Kutash K, Duchnowski AJ, Green AL, Ferron J (2013). Effectiveness of the parent Connectors program: results from a randomized controlled trial. School Mental Health: A Multidisciplinary Research and Practice Journal.

[75] Robbins V, Johnston, J., Barnett, H., Hobstetter, W., Kutash, K., Duchnowski, A. J., & Annis, S. Parent to parent: A synthesis of the emerging literature.: Tampa, FL: University of South Florida, The Louis de laParte Florida Mental Health Institute, Department of Child & Family Studies.; 2008.

[76] O’Connell MJ, Flanagan EH, Delphin-Rittmon ME, Davidson L. Enhancing outcomes for persons with co-occurring disorders through skills training and peer recovery support. J Ment Health. 2020;29(1):6–11. 10.1080/09638237.2017.1294733 28282996

[77] Galloway A, Pistrang N. “We’re stronger if we work together”: experiences of naturally occurring peer support in an inpatient setting. Journal of Mental Health. 2019;28(4):419–26. 10.1080/09638237.2018.1521925 30328759

[78] Tierney S, Bivins R, Seers K. Compassion in nursing: Solution or stereotype? Nurs Inq. 2019;26(1):e12271. 10.1111/nin.12271 PMC6492101 30548117

