# Supplementary material for: Understanding the support needs of parents of children with obsessive-compulsive disorder: a qualitative descriptive study in the UK
Source: BMC Psychiatry. 2023 May 3;23:309. doi: 10.1186/s12888-023-04637-8 (PMC10155140; doi:10.1186/s12888-023-04637-8)
Supplement: Supplementary file 1 — Supplementary Material 1 [file 12888_2023_4637_MOESM1_ESM.docx]

**Additional file 1: Topic guides for parental carers and professionals**

- Aim- to provide a detailed understanding of parental carer needs/challenges and preferences for support from both the parental carer and professional perspective
- Preamble to include the aim of the study, explanation of ethics, consent, confidentiality of interview, audio-recording, the structure of interview/focus group, and possible overlap of questions prior to beginning questions.
- This topic guide forms an outline of the possible questions and prompts that will be used flexibly and adapted and ordered according to the needs/preferences of each interview/focus group and insights from ongoing analysis. The ordering of these questions is not fixed, and questions will follow participants' narratives of their experiences and challenges when caring for a child or young person with OCD.

**Parental carer topic guide**

| **To explore** | **Questions** | **Prompts** |
| --- | --- | --- |
| **Support needs/challenges during the diagnostic process** | Can you tell me how you came to understand your child was experiencing symptoms of OCD? | Who did you initially turn to for support/advice? |
| **Accessing support** | Can you tell me how you came to be involved with services (e.g., GP, school, self-referral, private care)?    Can you tell me about any support you have had while looking after your son/daughter with OCD? | Can you tell me what gaining access to this service was like? How easy/difficult was it to access?  Can you tell me who helped/supported your son/daughter/family before these services were involved?  Was any signposting available or would have been helpful at this time  Explore- family/friends, online support, accessing charity resources  Explore- what it was like gaining access to these services |
| **Parental needs/coping strategies** | Can you tell me about anything that you think might help you feel more prepared/ able to cope with supporting your child with OCD? | Explore information needs/ uncertainty/peer support |
| **Current needs/challenges** | It would be very helpful if you could talk me through what your day involves supporting your child with OCD.  Can you tell me about any challenges you have faced this week?  Are there any things that have made these challenges more difficult/easier? | Prompt for practical and emotional burden, coping strategies  The researcher will encourage the parent to use the journal as an aid in exploring the challenges faced and their experiences, support needs and preferences.  How they felt, and what actions were taken,  What help did they seek or was available, and what help would they have ideally liked? |
| **Perceptions of child/family services** | Can you tell me about who/what service (s) is currently involved (or not involved/ waiting to be involved) in supporting your son/daughter and family?  It would be helpful if you could tell me what this service/support involves for you as a family.  Can you tell me how (or if) you have been involved with these service(s)? | Pattern/frequency of use of the service  Who attends  What different professional groups have been involved, e.g., schools/teachers  Pick up any negative or positive impact of engagement (what was helpful/unhelpful) for self and wider family  Suggestions about what would be most helpful |
| **Preferences and priorities for support** | Do you think your support needs have changed over time?  Can you tell me what you feel would be the most helpful for you right now as a parent- the ideal support? | Prompt for when support needs felt more or less pressing  If a parent/carer in a similar position approached you for advice, what might you tell them?  Explore – what this would involve and how it would be delivered. |
| **Final Question** | Is there anything else you would like to share that may help us understand ways to improve the support provided to parents of children with OCD, which we haven't discussed already? |  |

**Professional Topic Guide**

| **To explore** | **Questions** | Prompts |
| --- | --- | --- |
| **Perceptions of current services offered to families with a child who has OCD** | Could you begin by telling us what services are available in your organisation for families with OCD?  In your experience, how parents/carers are involved in these services?  How do you feel about this  What challenges do you face when working with families with a child who has OCD? Are there any areas of the service for families with OCD that you like to see developed or changed | Can you tell me a little bit about how it happens in practice  How do you feel about this?  Is it something that always happens, or not much ( if so, why not) It would be really useful to get your views on any sticking points concerning parent involvement?  What do you think currently works well around involving parents/carers?  Possible ways to improve the service for parental carers |
| **Parent needs** | In your experiences, what are the most pressing needs for parents/carers?  In your experiences, what strategies/resources have you found helpful in addressing/supporting parents' needs?  Could we ask what you have found the most successful and why? | e.g., family/friends, online support, charity resources, and peer support.  Do you have any examples of forms of support (if any) that haven't been successful and why? (or what they think WOULDN'T be helpful) |
| **Perceptions of service provision & parental support** | In your service provision (is it meaningful or), do you have the resources/remit to specifically identify and address parents' needs? | Can you tell me about these resources currently available (including informational resources)  How helpful have professionals or parents found these? |
|  | Do you have any suggestions about how the needs of parents/carers could be better met? | If relevant, could you compare 'ideal world' vs 'realistic'? |
|  | Have you got any thoughts on where parent support logically fits within services? |  |
| **Potential barriers to accessing support** | It would be helpful if you could talk through how families typically access your services? | What helps parents access support? (Or how could access be improved?)  Do you have any ideas about what gets in the way of parents' access to support? (e.g. organisational or individual barriers) |
| **Content of acceptable, feasible support** | It would be helpful if you could tell us your priorities as providers in terms of supporting parents?  What do you think parents' priorities are?  What do you think the ideal support for parents would look like  What do you think is currently feasible or realistic? | Content delivery, the format of support? |
| **Final Question** | Is there anything else you would like to share that may help us understand ways to improve the support provided to parents of children with OCD, which we haven't discussed already? |  |
