# Supplementary material for: Understanding the support needs of parents of children with obsessive-compulsive disorder: a qualitative descriptive study in the UK
Source: BMC Psychiatry. 2023 May 3;23:309. doi: 10.1186/s12888-023-04637-8 (PMC10155140; doi:10.1186/s12888-023-04637-8)
Supplement: Supplementary file 2 — Supplementary Material 2 [file 12888_2023_4637_MOESM2_ESM.docx]

**Additional file 2: The analytical process**

| **Definition** | **Purpose** | **Process** |
| --- | --- | --- |
| Stage 1  Familiarisation | To become fully acquainted with the data To identify what people are saying that is relevant to the research objective | Reading and re-reading transcripts & listening to audio files & checking accuracy & ambiguities  Making handwritten notes on transcripts  Reading field notes and making case summaries  Identifying key ideas within individual accounts |
| Stage 2  Development of thematic framework | A formal system to manage and organise the data and to allow systematic and comprehensive coverage and comparison of the data-set | To compare and contrast data between and across cases  Uploading of transcripts to qualitative data analysis software QSR NVivo 12  Grouping of data according to descriptive codes  Analytical memo writing of key ideas  Returning to stage 1 where necessary |
| Stage 3  Indexing and sorting the data | To identify dominant recurrent unifying ideas/concepts occurring across the data concerning the research objective | Applying thematic framework to the data  Recode or refine dimensions of existing framework according to developing analytical insights  Returning to stage 1 and 2 where necessary |
| Stage 4  Charting data | To summarize and display the data and to begin to distill overarching themes. | To create a table based on the thematic framework  To summarize the data within the table.Continue cross-examination of the data  Returning to stage 1 and 2 where necessary |
| Stage 5  Describing and interpreting the data | To actively look for meaning in the data to explain key features and patterns found across the data set concerning the research objective  To introduce theoretical concepts or theories in as far as they actually match the data | To identify overarching themes and sub-themes which convey across the data and which relate to the research objective |

Adapted from: Ritchie, J., Lewis, J., Nicholls, C. M., & Ormston, R. (2013). Qualitative research practice: A guide for social science students and researchers: sage.
